# Treatment-Emergent Epiretinal Membrane in Diabetic Macular Edema: A Real-World OCT-Based Comparative Pharmacotherapy Study Across Intravitreal Agents—The TRIDENT-DME Study

**DOI:** 10.3390/ph19091390

**Published:** 2026-09-02

**Authors:** Debdulal Chakraborty, Tushar Kanti Sinha, Rupak Kanti Biswas, Aniruddha Maiti, Manab Jyoti Barman, Ranabir Bhattacharya, Subhendu Kumar Boral, Arnab Das

**Affiliations:** 1 Disha Eye Hospitals, Barrackpore, Kolkata 700120, West Bengal, India; 2 Nethralayam Superspeciality Eye Hospital, Kolkata 700099, West Bengal, India; 3 Global Eye Hospitals Kolkata, Kolkata 700106, West Bengal, India; 4 ASG Eye Hospitals, Guwahati 781001, Assam, India

**Keywords:** diabetic macular edema, epiretinal membrane, dexamethasone implant, anti-VEGF, ranibizumab, bevacizumab, aflibercept, faricimab, optical coherence tomography

## Abstract

**Purpose**: To evaluate the incidence, predictors, and clinical impact of treatment-emergent epiretinal membrane (ERM) in diabetic macular edema (DME) treated with dexamethasone implant, aflibercept, ranibizumab, bevacizumab, or faricimab. **Methods**: In this multicenter, retrospective, nonrandomized study, 501 eyes with center-involving DME were assessed and 423 eyes without ERM on pretreatment OCT and with 24-month follow-up were included. Eyes were classified by the intravitreal agent selected by the treating physician in routine care and maintained throughout follow-up; eyes requiring a drug switch or retinal laser/vitrectomy during follow-up were excluded. The last eligible pre-index SD-OCT confirmed the absence of ERM; BCVA, central subfield thickness (CST), and vitreomacular-interface (VMI) morphology were assessed at baseline and 6, 12, 18, and 24 months. Crude comparisons, multivariable logistic regression, and Kaplan–Meier/log-rank analyses were performed. **Results**: ERM developed in 36/423 eyes (8.5%). Incidence was 16.0% with dexamethasone implant, 7.5% with aflibercept, 7.7% with ranibizumab, 7.4% with bevacizumab, and 4.3% with faricimab. Dexamethasone implant showed higher ERM odds than pooled non-dexamethasone therapy after adjustment (OR 2.24, 95% CI 1.02–4.92; *p* = 0.044). Baseline VMI abnormality, severe NPDR/PDR, prior PRP, and higher baseline CST were also associated with ERM. Eyes developing ERM had smaller BCVA gains (+2.8 vs. +7.1 ETDRS letters; *p* < 0.001) and CST reductions (88.6 vs. 130.8 µm; *p* = 0.004). **Conclusions**: Treatment-emergent ERM was associated with baseline ocular characteristics and treatment category and with poorer 24-month outcomes. These observational drug-group signals require prospective confirmation.

## 1. Introduction

Diabetic macular edema (DME) remains a leading cause of visual impairment in patients with diabetic retinopathy (DR), and its management has been transformed by intravitreal pharmacotherapy. For center-involving DME with visual impairment, anti-vascular endothelial growth factor (anti-VEGF) therapy is generally the preferred initial treatment. Ranibizumab, an antibody fragment directed against VEGF-A, has an established evidence base for improving visual acuity and DR severity and is used with loading, pro re nata, or treat-and-extend regimens. Aflibercept is a soluble decoy receptor that binds VEGF-A, VEGF-B, and placental growth factor and is frequently selected when baseline visual acuity is relatively poor, retinal thickening or fluid burden is substantial, or rapid anatomical control is desired. Bevacizumab, a full-length anti-VEGF antibody used off-label for DME, remains an important lower-cost option, particularly in resource-constrained settings [1]. Intravitreal dexamethasone 0.7 mg implant provides sustained corticosteroid delivery and targets inflammatory and vascular-permeability pathways; it is commonly used for persistent or recurrent DME, an incomplete response to anti-VEGF therapy, pseudophakic or vitrectomized eyes, or when a lower injection frequency is desirable and intraocular-pressure monitoring is feasible [2,3]. Faricimab, a bispecific antibody that inhibits both VEGF-A and angiopoietin-2, offers an additional first-line or switch option when enhanced durability and vascular stabilization are treatment priorities [4].

Comparative effectiveness depends on the baseline phenotype and the outcome being prioritized rather than on a single drug hierarchy. In DRCR Retina Network Protocol T, aflibercept, bevacizumab, and ranibizumab all improved vision; outcomes were similar when baseline visual acuity was 20/32 to 20/40, whereas among eyes with baseline visual acuity of 20/50 or worse, aflibercept produced greater two-year visual improvement than bevacizumab, but not ranibizumab [1]. Protocol AC subsequently showed that a bevacizumab-first strategy with protocol-directed switching could achieve a similar mean two-year visual outcome to aflibercept monotherapy, although 70% of bevacizumab-first eyes required conversion to aflibercept [5]. Dexamethasone implant improves visual and anatomical outcomes and reduces injection frequency in appropriately selected eyes, but its use is limited by cataract progression and ocular hypertension [2,3]. Newer durability data have further broadened treatment choice: in PHOTON, aflibercept 8 mg administered at 12- or 16-week intervals after loading maintained visual and anatomical outcomes through 96 weeks that were non-inferior to aflibercept 2 mg every 8 weeks, with similar safety [6]. In YOSEMITE and RHINE, faricimab achieved visual gains comparable with aflibercept 2 mg while providing greater anatomical drying and individualized extension up to every 16 weeks; the four-year RHONE-X extension subsequently showed sustained gains, with approximately 80% of patients receiving faricimab at intervals of at least 12 weeks by study completion [4,7].

Accordingly, no agent can be designated as universally most effective for all eyes with DME. Aflibercept has the strongest direct comparative advantage over bevacizumab in eyes with worse baseline vision; ranibizumab remains a well-established effective anti-VEGF option; bevacizumab offers major economic accessibility but may require switching in eyes with an inadequate response; faricimab and high-dose aflibercept provide contemporary durability advantages; and dexamethasone implant remains valuable for selected inflammatory, refractory, pseudophakic, or vitrectomized phenotypes despite its lens- and pressure-related risks. This phenotype-driven selection is especially relevant to observational comparisons because treatment assignment in routine practice is closely linked to baseline disease severity, prior response, ocular comorbidity, follow-up feasibility, and cost.

Although DME is primarily considered a disorder of vascular permeability and inflammation, the vitreomacular interface may influence both disease course and treatment response. Epiretinal membrane (ERM), vitreomacular adhesion, vitreomacular traction, posterior hyaloid abnormalities, and vitreoschisis may coexist with DME and may contribute to persistent retinal thickening through tractional forces, altered retinal biomechanics, impaired drug diffusion, and maintenance of a chronic inflammatory or pro-fibrotic microenvironment [8,9]. Careful OCT-based assessment of the vitreomacular interface is therefore clinically relevant during long-term DME management.

Treatment-emergent ERM in DME has recently gained attention as a clinically meaningful vitreomacular-interface outcome during intravitreal therapy. Emerging evidence suggests that ERM may develop during follow-up in a subset of treated DME eyes and may be associated with a less favorable anatomical response [10,11]. However, ERM formation should not automatically be interpreted as a direct drug-specific adverse effect because it may reflect overlapping influences from baseline DR severity, chronicity of macular edema, prior retinal laser exposure, vitreomacular-interface status, inflammatory and fibrotic activity, and cumulative treatment exposure.

The relationship between cumulative intravitreal administration count and ERM formation is particularly important in real-world practice. Eyes requiring more injections or implants may have more persistent edema, greater ischemic drive, more severe DR, or a more active inflammatory-fibrotic milieu. Conversely, repeated intravitreal procedures may theoretically influence the posterior vitreous cortex and vitreomacular interface [12]. Comparative real-world analyses should therefore consider baseline disease severity, CST, prior PRP or macular laser, lens status, prior vitrectomy, previous intravitreal exposure, the fixed 24-month administration count, and baseline VMI abnormalities. Administration count denotes procedures over time and is not a pharmacologic dose-equivalence measure across agents.

Despite these emerging observations, limited real-world evidence compares treatment-emergent ERM across dexamethasone implant, aflibercept, ranibizumab, bevacizumab, and faricimab within a unified OCT-based framework. Such an analysis addresses a comparative ocular-pharmacotherapy and drug-safety question while recognizing treatment-selection confounding. The purpose of the present TRIDENT-DME study (Treatment-related Retinal Interface changes and Development of Epiretinal membrane across iNtravitreal Therapies in Diabetic Macular Edema) was to evaluate the incidence, timing, predictors, and clinical impact of treatment-emergent ERM in DME treated with contemporary intravitreal agents.

## 2. Results

### 2.1. Participant Flow

**Figure 1:**
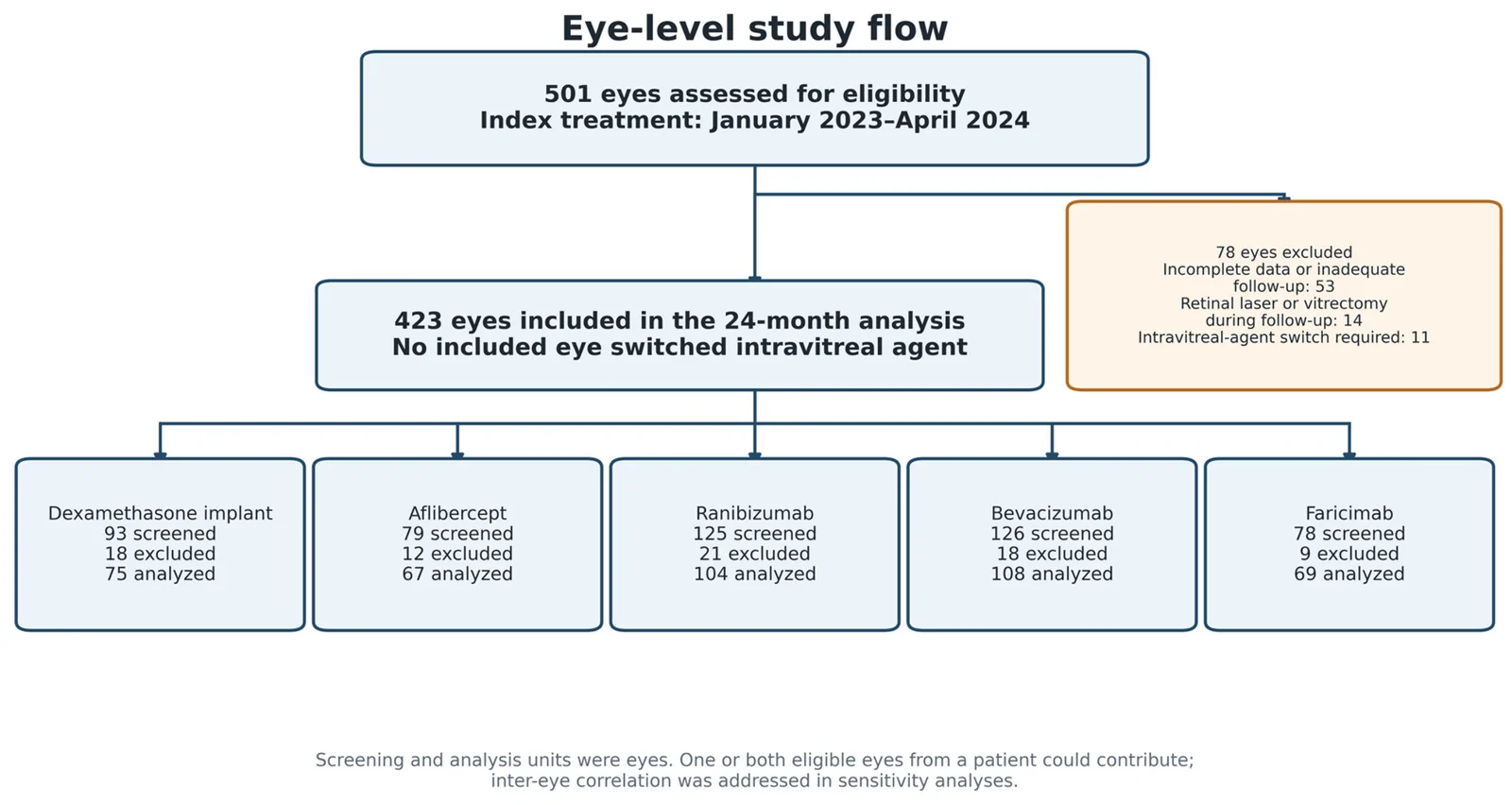
Eye-level study flow diagram. Exclusions and analyzed-eye counts are shown overall and by intravitreal-agent group. One or both eligible eyes from a patient could contribute; inter-eye correlation was addressed in sensitivity analyses.

Screening and analysis were performed at the eye level. Of 501 eyes assessed for eligibility, 78 were excluded: 53 because required clinical/OCT data or adequate 24-month follow-up were unavailable, 14 because retinal laser or vitrectomy was performed during follow-up, and 11 because an intravitreal-agent switch was required. The final analysis included 423 eyes; no included eye switched treatment. Group-specific screening and inclusion counts are shown in Figure 1.

### 2.2. Study Population and Baseline Characteristics

The final analysis comprised 423 eyes with DME: 75 treated with dexamethasone implant, 67 with aflibercept, 104 with ranibizumab, 108 with bevacizumab, and 69 with faricimab. All included eyes had no clinically evident macular ERM on the pretreatment baseline OCT and completed the required 24-month follow-up.

The mean age of the study population was 60.8 ± 9.3 years, and 238 eyes were from male patients (56.3%). Mean baseline BCVA was 0.65 ± 0.23 logMAR, corresponding to approximately 52.4 ± 11.4 ETDRS letters. Mean baseline CST was 474.5 ± 86.9 µm. Baseline anatomical and clinical features differed across treatment groups, with dexamethasone implant-treated eyes showing higher baseline CST and longer DME duration. Treatment-naive eyes comprised 97/423 eyes (22.9%), while 326/423 eyes (77.1%) had received prior intravitreal treatment before the index date. Treatment-naive eyes accounted for 12/75 eyes (16.0%) in the dexamethasone implant group, 11/67 eyes (16.4%) in the aflibercept group, 27/104 eyes (26.0%) in the ranibizumab group, 30/108 eyes (27.8%) in the bevacizumab group, and 17/69 eyes (24.6%) in the faricimab group.

Prior retinal laser exposure was also recorded because of its potential relationship with baseline disease severity and vitreomacular interface remodeling. Prior PRP was present in 164/423 eyes (38.8%), and prior macular laser was present in 127/423 eyes (30.0%). Forty-nine eyes (11.6%) had received both PRP and macular laser, while 181 eyes (42.8%) had no history of prior retinal laser. Baseline demographic and ocular characteristics are summarized in Table 1.

### 2.3. Treatment Exposure and DRSS Change

The 24-month intravitreal administration count differed across treatment groups (Table 2). Eyes treated with dexamethasone implant received a mean of 4.8 ± 1.9 implants. Eyes treated with aflibercept, ranibizumab, bevacizumab, and faricimab received 7.2 ± 1.4, 8.8 ± 1.4, 9.5 ± 1.4, and 6.8 ± 1.4 injections, respectively (*p* < 0.001). These are procedure counts over a fixed period and are not dose-equivalent across agents. Mean prior injection counts before the index date were 2.5 ± 1.9, 2.3 ± 1.8, 1.6 ± 1.4, 1.5 ± 1.3, and 2.0 ± 1.7, respectively (*p* < 0.001).

The distribution of DRSS change differed across treatment groups (*p* = 0.008). A two-step or greater DRSS improvement was observed in 5/75 eyes (6.7%) treated with dexamethasone implant, 15/67 eyes (22.4%) treated with aflibercept, 25/104 eyes (24.0%) treated with ranibizumab, 18/108 eyes (16.7%) treated with bevacizumab, and 19/69 eyes (27.5%) treated with faricimab. Stable DRSS was observed in 56/75 eyes (74.7%), 47/67 eyes (70.1%), 72/104 eyes (69.2%), 79/108 eyes (73.1%), and 47/69 eyes (68.1%), respectively. Two-step or greater DRSS worsening was observed in 14/75 eyes (18.7%), 5/67 eyes (7.5%), 7/104 eyes (6.7%), 11/108 eyes (10.2%), and 3/69 eyes (4.3%), respectively. This analysis was considered exploratory because DRSS change is a post-treatment variable.

### 2.4. Incidence of Treatment-Emergent ERM

**Figure 2:**
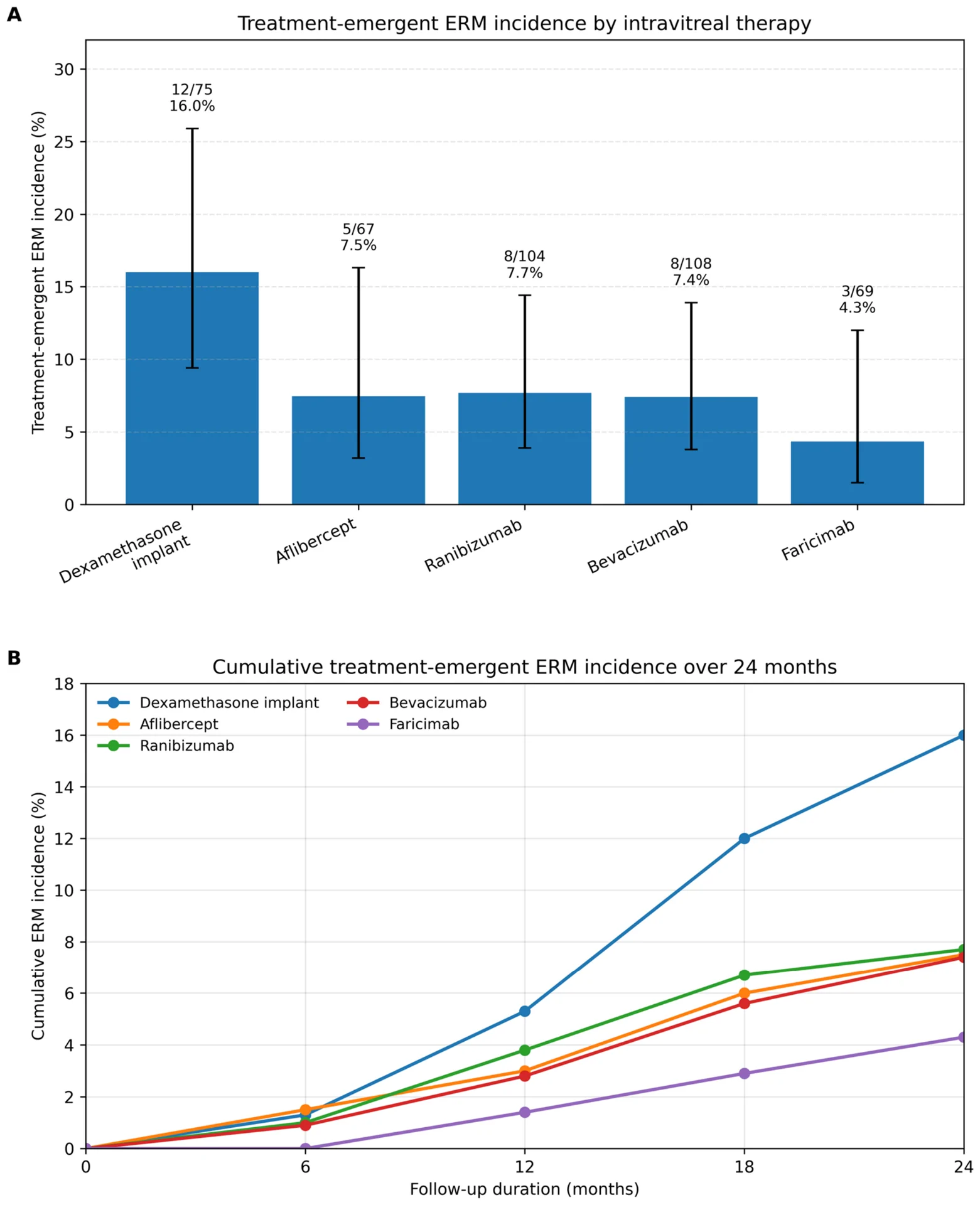
Treatment-emergent epiretinal membrane incidence according to intravitreal therapy. (**A**) Crude 24-month incidence with Wilson 95% confidence intervals; labels indicate events/eyes and percentages. (**B**) Cumulative incidence at 6, 12, 18, and 24 months. Separation was most apparent after 12 months, with the highest numerical incidence in the dexamethasone implant group. Comparisons shown are unadjusted.

During 24 months of follow-up, treatment-emergent ERM developed in 36/423 eyes (8.5%). ERM developed in 12/75 eyes (16.0%) treated with dexamethasone implant, 5/67 (7.5%) with aflibercept, 8/104 (7.7%) with ranibizumab, 8/108 (7.4%) with bevacizumab, and 3/69 (4.3%) with faricimab (Table 3; Figure 2A). Dexamethasone implant-treated eyes showed the highest numerical incidence; however, the overall five-group comparison was not statistically significant (χ^2^ = 7.29, df = 4, *p* = 0.121), and the 95% confidence intervals overlapped.

When dexamethasone implant-treated eyes were compared with all non-dexamethasone-treated eyes pooled together, ERM formation was more frequent in the dexamethasone implant group than in the pooled non-dexamethasone group (12/75, 16.0% vs. 24/348, 6.9%). This corresponded to a relative risk of 2.32 and an unadjusted odds ratio of 2.57 (95% CI 1.22–5.41; *p* = 0.020).

### 2.5. Pairwise and Anti-VEGF Subgroup Comparisons

Exploratory pairwise analysis showed numerically higher ERM incidence with dexamethasone implant than with each individual comparator group; however, after adjustment for multiple comparisons, individual pairwise comparisons were interpreted cautiously (Table 4). Among conventional anti-VEGF agents, ERM incidence was similar with aflibercept, ranibizumab, and bevacizumab (7.5%, 7.7%, and 7.4%, respectively; *p* = 0.997). However, when faricimab was included among non-dexamethasone therapies, it showed the lowest numerical ERM incidence.

### 2.6. Timing of ERM Development

**Figure 3:**
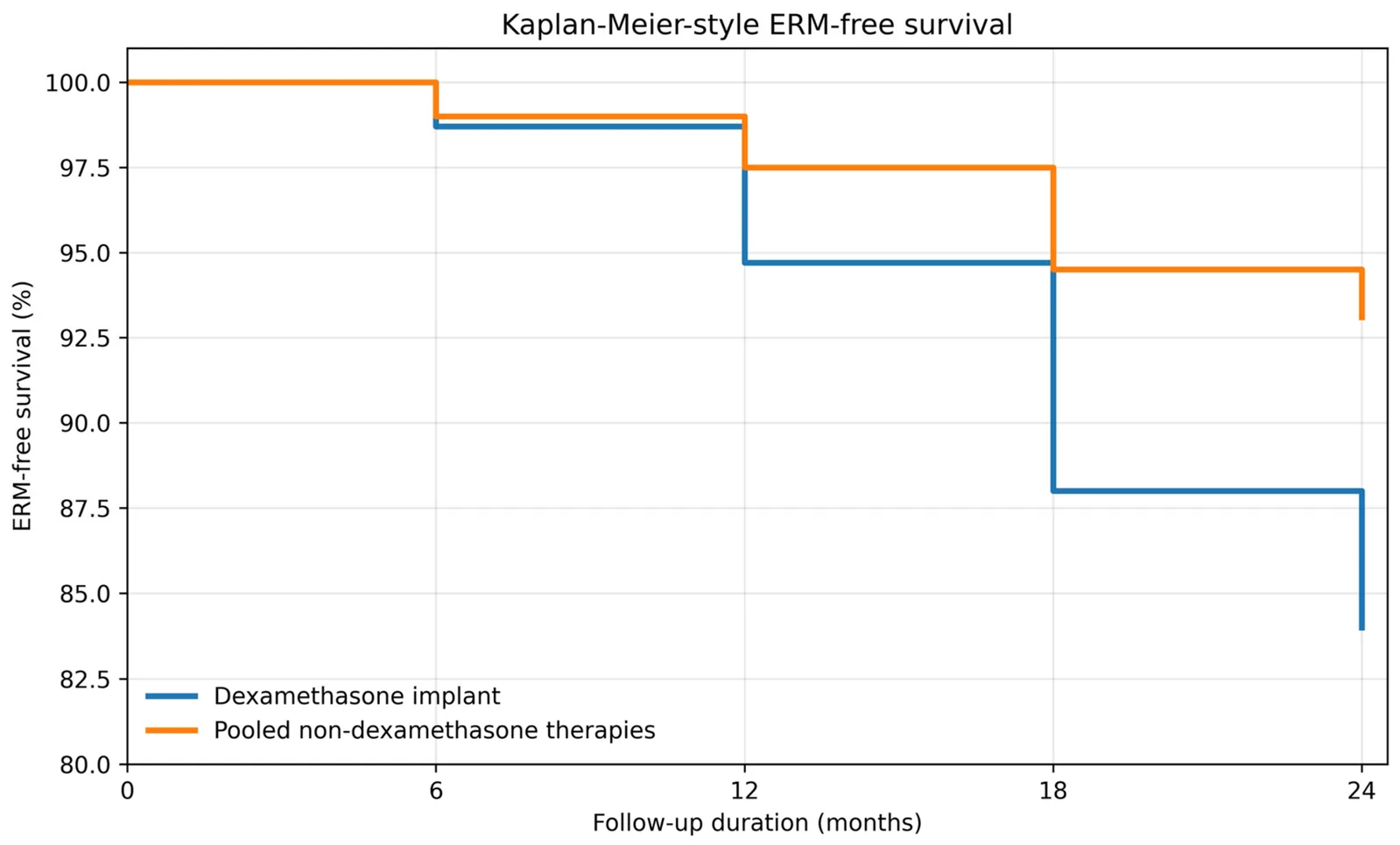
Kaplan–Meier estimate of ERM-free survival over 24 months for dexamethasone implant-treated eyes and pooled non-dexamethasone-treated eyes. ERM-free survival at 24 months was 84.0% and 93.1%, respectively (log-rank *p* = 0.010). The number-at-risk table reports eyes under observation immediately before each scheduled assessment.

ERM development occurred gradually during follow-up (Figure 2B). In dexamethasone implant-treated eyes, cumulative ERM incidence increased from 1.3% at 6 months to 5.3% at 12 months, 12.0% at 18 months, and 16.0% at 24 months. Temporal separation was most apparent after 12 months. By month 24, cumulative incidence remained below 8% in each conventional anti-VEGF group and below 5% in the faricimab group. Kaplan–Meier analysis showed 24-month ERM-free survival of 84.0% in the dexamethasone implant group and 93.1% in the pooled non-dexamethasone group (log-rank *p* = 0.010; Figure 3).

### 2.7. Univariate and Multivariable Analyses

**Figure 4:**
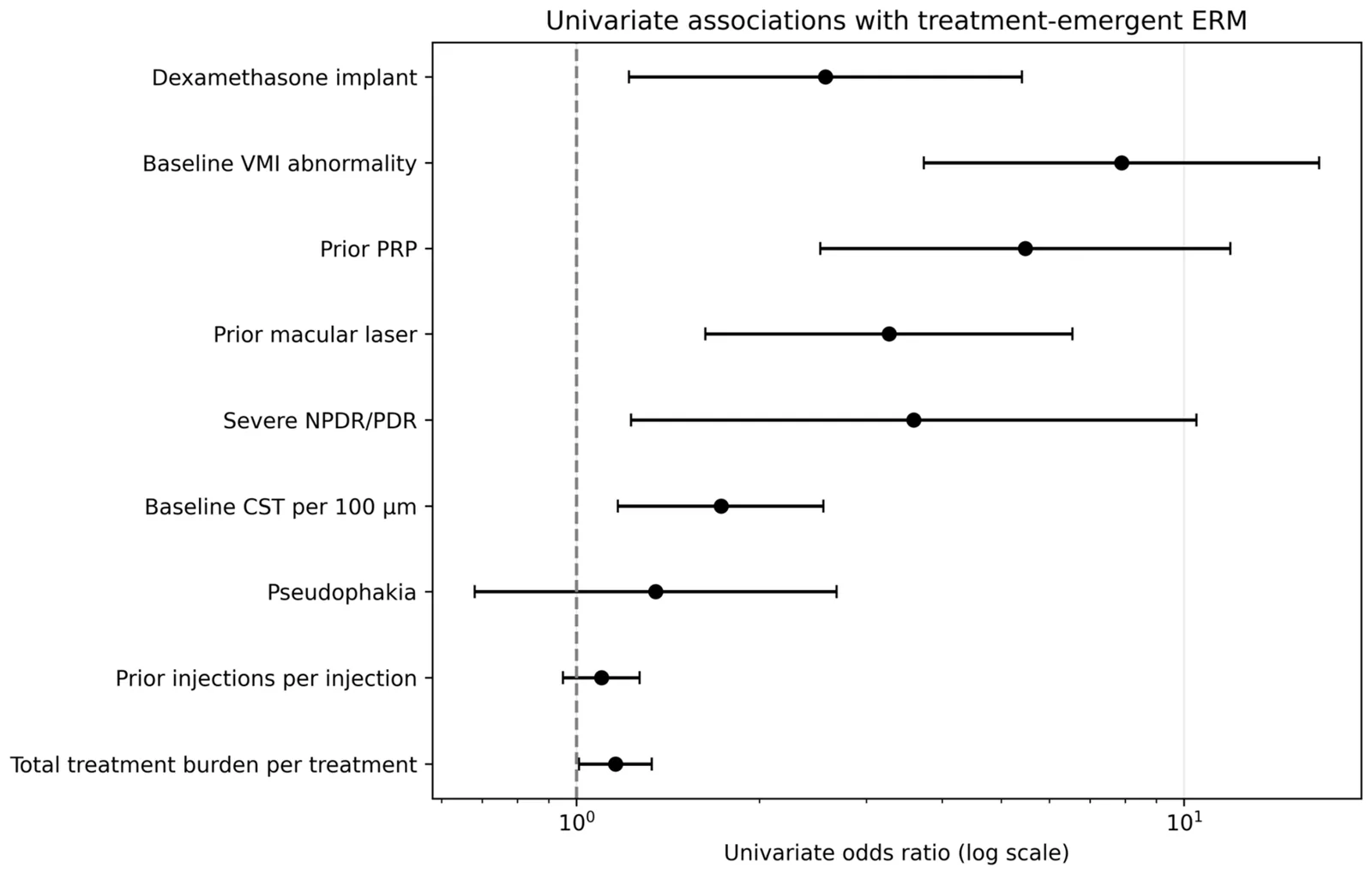
Forest plot of univariate associations with treatment-emergent ERM. Odds ratios are shown on a logarithmic scale. VMI abnormality, prior PRP, severe NPDR/PDR, higher baseline CST, and dexamethasone implant treatment were associated with higher ERM odds. The 24-month administration count represents procedure frequency, not drug dose.

Univariate analyses assessed clinically relevant variables, including age, sex, baseline BCVA, baseline CST, baseline DR severity, PDR status, prior PRP, prior macular laser, lens status, prior vitrectomy, prior injection count, 24-month intravitreal administration count, baseline VMI abnormality, and treatment category (Table 5; Figure 4). ERM formation was associated with dexamethasone implant treatment, worse baseline BCVA, higher baseline CST, severe NPDR/PDR, PDR, prior PRP, prior macular laser, administration count, and baseline VMI abnormality.

To reduce overfitting, the primary multivariable logistic model was restricted to prespecified covariates. Dexamethasone implant remained associated with treatment-emergent ERM compared with pooled non-dexamethasone therapy (adjusted OR 2.24, 95% CI 1.02–4.92; *p* = 0.044). Baseline VMI abnormality was the strongest predictor (adjusted OR 6.10, 95% CI 2.80–13.31; *p* < 0.001). Severe NPDR/PDR, prior PRP, and higher baseline CST were also associated with ERM (Table 6). The 24-month administration count was included only as a post-baseline exposure adjustment and was not independently significant.

### 2.8. Visual and Anatomical Outcomes According to ERM Status

**Figure 5:**
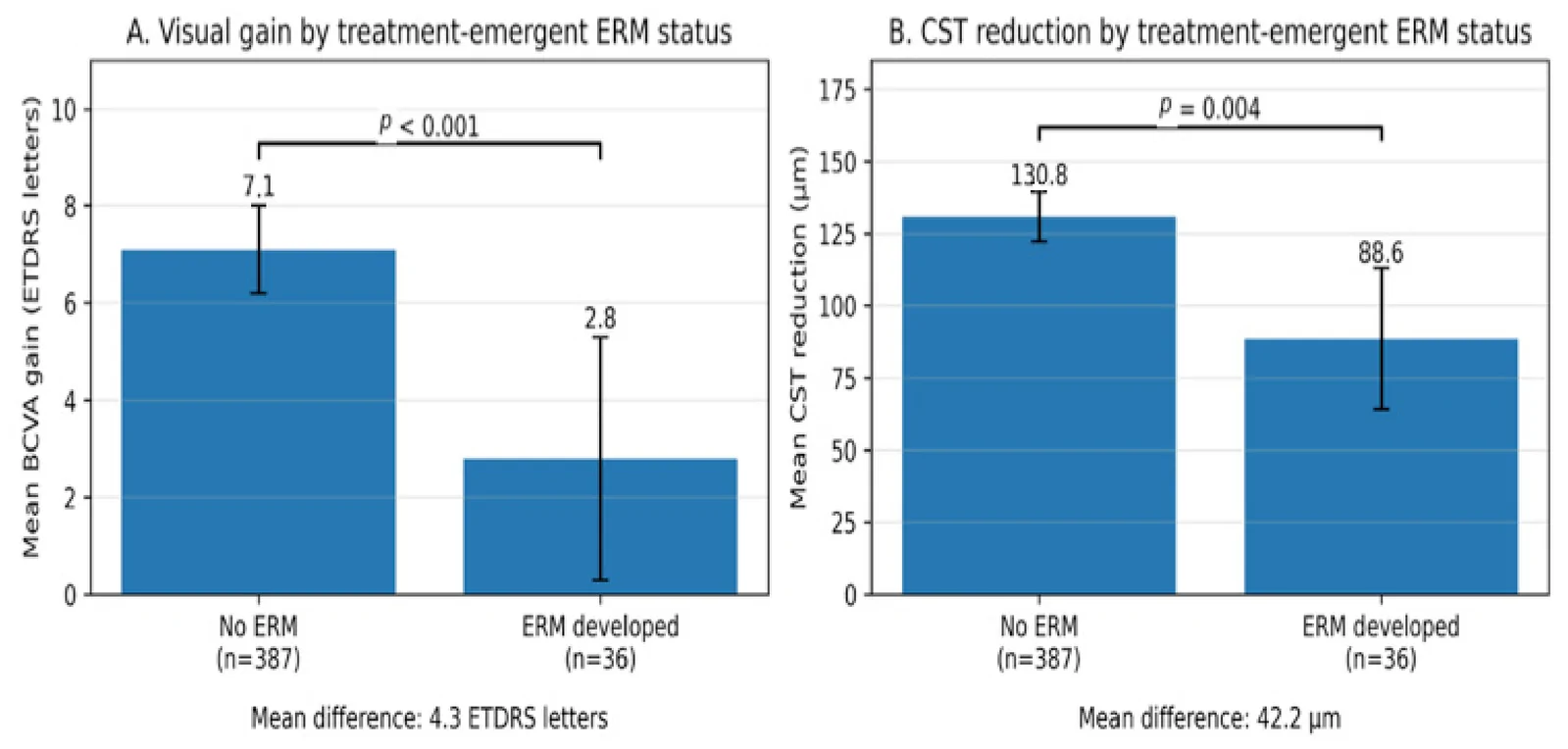
Clinical impact of treatment-emergent ERM. (**A**) Mean BCVA gain was lower in eyes that developed ERM than in eyes without ERM. (**B**) Mean CST reduction was lower in eyes that developed ERM. Error bars represent 95% confidence intervals.

Eyes that developed treatment-emergent ERM had less favorable visual and anatomical outcomes than eyes that did not develop ERM (Table 7). Mean BCVA gain was +7.1 ETDRS letters without ERM and +2.8 letters with ERM (mean difference 4.3 letters; *p* < 0.001). Mean CST reduction was 130.8 µm without ERM and 88.6 µm with ERM (mean difference 42.2 µm; *p* = 0.004) (Figure 5). After adjustment, ERM formation remained associated with worse final BCVA and higher final CST.

### 2.9. Sensitivity and Secondary Analyses

Secondary and sensitivity analyses evaluated prior macular laser, lens status, prior vitrectomy, prior injection count, treatment-naive eyes, individual anti-VEGF agent, DRSS change, and exclusion of eyes with baseline VMA/VMT. The dexamethasone implant effect generally remained directionally consistent, although it was attenuated after excluding baseline VMA/VMT, supporting the importance of baseline VMI status (Table 8).

### 2.10. Summary of Principal Findings

**Figure 6:**
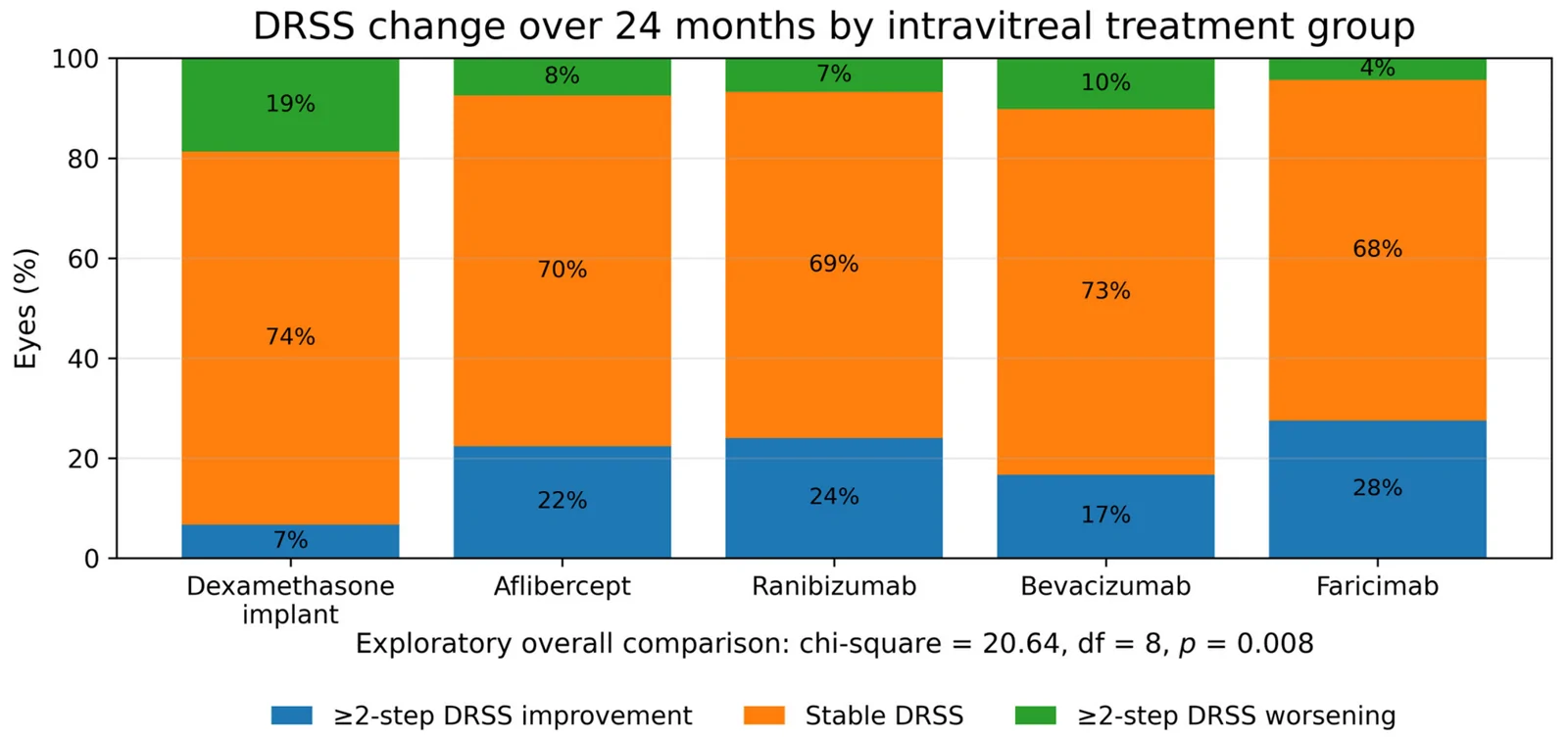
Distribution of DRSS change by intravitreal treatment group. Bars show the proportion of eyes with at least two-step DRSS improvement, stable DRSS, or at least two-step DRSS worsening. This analysis was exploratory because DRSS change is a post-treatment variable.

In this real-world OCT-based comparative analysis, treatment-emergent ERM developed in 8.5% of DME eyes over 24 months. Dexamethasone implant-treated eyes showed the highest numerical incidence and higher crude and adjusted ERM odds than pooled non-dexamethasone therapies. Conventional anti-VEGF agents showed comparable crude rates. Faricimab showed the lowest numerical ERM incidence. and the highest numerical proportion of two-step or greater DRSS improvement, but these findings were exploratory. DRSS change by treatment group is shown in Figure 6.

## 3. Discussion

The principal findings of the present study were that treatment-emergent ERM developed in 8.5% of eyes with DME over 24 months and was associated with less favorable visual and anatomical outcomes. Within this cohort, dexamethasone implant-treated eyes had the highest numerical ERM incidence and a significantly higher crude and adjusted ERM risk than pooled non-dexamethasone-treated eyes. Aflibercept, ranibizumab, and bevacizumab had similar crude ERM rates, whereas faricimab had the lowest numerical rate. These results provide the basis for the interpretations below, which are considered separately alongside previously published evidence.

The higher ERM rate observed in dexamethasone implant-treated eyes in the present cohort is directionally consistent with previously published observational evidence. Kang et al. and Chang et al. independently reported secondary ERM formation during DME treatment, with a higher ERM incidence after dexamethasone implant than after anti-VEGF therapy and a less favorable anatomical response after ERM development [10,11]. The present study extends those reports by evaluating dexamethasone implant, aflibercept, ranibizumab, bevacizumab, and faricimab within one real-world OCT-based framework. Multicenter evidence has also shown that baseline OCT biomarkers are associated with functional outcomes after dexamethasone implant, emphasizing the importance of retinal phenotype when treatment results are interpreted [11,12]. None of these data establish that dexamethasone directly causes ERM. In routine practice, dexamethasone implant is frequently selected for eyes with chronic or previously treated DME, greater retinal thickening, inflammatory features, pseudophakia, or limited feasibility for frequent follow-up [2,3]. We therefore interpret the association observed in our cohort as potentially reflecting treatment selection, baseline disease severity, VMI susceptibility, chronic inflammation, and treatment-related factors rather than a simple drug-attributable adverse effect.

In the present study, baseline VMI abnormality was the strongest predictor of treatment-emergent ERM in the adjusted model. In addition, the dexamethasone association was attenuated when eyes with baseline VMA or VMT were excluded. These are findings from our cohort and suggest that pre-existing posterior hyaloid or VMI alterations may identify eyes predisposed to subsequent preretinal fibrocellular proliferation. Separately, previous OCT-based studies have shown that VMI abnormalities can influence treatment response in DME and may develop during anti-VEGF therapy [13,14,15,16,17,18,19,20,21]. Experimental work using an in vitro ERM model further suggests that a preretinal membrane can alter antibody permeability, although this mechanism has not been demonstrated in vivo in the present cohort [22]. Clinical studies of ranibizumab-treated DME have reported less favorable visual or anatomical responses in eyes with ERM or other VMI abnormalities [23,24]. The DRCR.net vitrectomy study also demonstrated that releasing vitreomacular traction in selected DME eyes commonly reduced retinal thickening, although visual outcomes were variable [25,26]. More recently, Maguire et al. reported a high prevalence of VMI abnormalities in newly diagnosed center-involving DME and later separation of visual and anatomical responses during anti-VEGF treatment [26], while a 2023 systematic review and meta-analysis concluded that VMI configuration modifies anti-VEGF treatment outcomes in DME [27]. On the basis of our results and this independent evidence, we interpret systematic OCT assessment of the VMI before and during treatment as clinically relevant, particularly in eyes with chronic or recurrent edema. Proposed mechanisms such as tangential traction, altered retinal biomechanics, and impaired fluid resolution remain biological explanations rather than mechanisms directly demonstrated by the present study.

Within our adjusted analysis, severe NPDR/PDR status, higher baseline CST, and prior PRP were independently associated with treatment-emergent ERM. We interpret prior PRP primarily as a marker of more advanced ischemic diabetic retinopathy rather than as evidence that laser treatment directly causes ERM. Similarly, higher baseline CST may represent greater anatomical burden, longer-standing edema, or more active leakage and inflammation. Previous studies have independently associated ERM in diabetic eyes with greater DR severity and cumulative anti-VEGF exposure and have described the clinical impact of ERM in DME [12,15]. Thus, the relationship between disease severity and ERM observed in our cohort is consistent with prior evidence, although the present retrospective analysis cannot establish the underlying causal pathway.

In the present dataset, the fixed 24-month intravitreal administration count was associated with ERM in univariate analysis but was not independently significant after adjustment for treatment category, baseline VMI status, DR severity, prior PRP, and baseline CST. This finding suggests that procedure count may partly mark disease severity, chronicity, or incomplete response. Independent observational work has also linked cumulative anti-VEGF exposure with ERM in diabetic eyes [12]. Neither that report nor our analysis demonstrates that repeated procedures cause ERM. Moreover, the count is a post-baseline exposure accumulated over the same 24-month outcome window and cannot be interpreted as a drug-dose effect; reverse causation is possible because eyes with persistent disease may receive more treatment. We therefore use the term administration count and interpret it as part of a disease–treatment interaction.

Within the present cohort, crude ERM incidence was nearly identical with aflibercept, ranibizumab, and bevacizumab (7.5%, 7.7%, and 7.4%, respectively; *p* = 0.997). Accordingly, within the limits of this study, we interpret baseline disease characteristics and VMI status as potentially more influential than the specific conventional anti-VEGF molecule in determining ERM risk in this dataset. The absence of a detected difference should not be interpreted as proof that the three agents have identical biological effects because the study was not powered to exclude small molecule-specific differences. This interpretation is particularly relevant because chronicity, previous laser exposure, baseline DR severity, and prior treatment history were heterogeneous within our real-world cohort.

In the present study, faricimab-treated eyes had the lowest numerical ERM incidence and the highest numerical proportion of two-step or greater DRSS improvement; neither observation established superiority, and both were exploratory. Independently, the post hoc YOSEMITE/RHINE analysis reported ERM in 3.8% of eyes receiving faricimab every 8 weeks and 7.6% receiving aflibercept every 8 weeks through 100 weeks (OR 0.48, 95% CI 0.29–0.81), corresponding to a 52% lower reported risk with faricimab in that comparison [16]. Faricimab inhibits VEGF-A and angiopoietin-2, a pathway implicated in vascular instability, permeability, inflammation, and fibrosis [4,17]. Differences in treatment selection and prior exposure may have contributed to our lower numerical faricimab signal; it therefore remains hypothesis-generating.

The DRSS results reported here were findings of the present exploratory analysis. Two-step or greater DRSS improvement was numerically more frequent in faricimab-, ranibizumab-, and aflibercept-treated eyes than in dexamethasone implant-treated eyes, whereas DRSS worsening was numerically more frequent in the dexamethasone group. Previous clinical trial analyses have independently demonstrated that anti-VEGF therapy can improve DR severity in eyes with DME [18,19,20]. We hypothesize that greater suppression of vascular leakage, neovascular drive, and ischemia-related activity could modify a pro-fibrotic retinal environment, but this pathway was not evaluated in our study. Moreover, DRSS change is a post-treatment variable that may lie on the causal pathway between treatment, disease modification, and ERM development. It was therefore excluded from the primary multivariable model and analyzed separately. Any relationship between DRSS regression and reduced ERM risk remains speculative pending prospective studies using standardized DRSS assessment.

In the present study, eyes that developed ERM had smaller BCVA gains and less CST reduction than eyes that remained ERM-free. ERM formation also remained associated with worse final BCVA and higher final CST after adjustment. These outcome differences are findings from our cohort and support the clinical relevance of treatment-emergent ERM. We interpret ERM as potentially contributing to persistent thickening and limited visual recovery through tractional and structural effects, although the retrospective design cannot prove that ERM caused the poorer outcomes. This interpretation is consistent with independent clinical reports in which ERM or other VMI abnormalities were associated with reduced visual or anatomical response during DME treatment [9,13,15,23,24,26,27]. It is also biologically compatible with the experimental ERM permeability model described by Namba et al. [22]. In clinical practice, our findings support careful reassessment of the VMI when a new ERM is detected, including consideration of the relative contributions of tractional and vascular components to residual edema.

The ERM findings should also be considered within the broader evidence base for DME treatment. Protocol T demonstrated that aflibercept, bevacizumab, and ranibizumab all improve vision in vision-impairing DME, with relative differences influenced by baseline visual acuity [1]. Protocol V subsequently showed that, in center-involved DME with good baseline visual acuity, initial observation or focal/grid laser with aflibercept reserved for visual acuity worsening can be reasonable [28]. Phase III RISE/RIDE and VISTA/VIVID studies established sustained efficacy of ranibizumab and aflibercept, respectively [29,30]. Randomized BEVORDEX data compared bevacizumab and dexamethasone implant [31], whereas Protocol AC evaluated bevacizumab-first step therapy with protocol-directed switching to aflibercept [5]. Newer phase III programs have extended efficacy and durability evidence to faricimab and aflibercept 8 mg [4,6,7]. These trials define efficacy, safety, and administration frequency, but ERM formation was not their primary endpoint. The present study addresses a distinct VMI outcome and should not be used to rank the overall efficacy of these agents.

The numerical faricimab signal also has pharmacological plausibility, although it is not clinical proof of ERM prevention. In retinal neovascularization and ischemia–reperfusion models, dual Ang-2/VEGF-A inhibition reduced leakage and inflammatory-cell accumulation and showed complementary anti-inflammatory and neuroprotective effects [32]. In a 2026 in vitro study of primary human retinal pericytes, faricimab reduced pericyte-to-myofibroblast transition, migratory capacity, and fibrotic-marker expression [33]. ERM is a complex fibrocellular process, and these preclinical/in vitro findings support a testable mechanism rather than establish that faricimab prevents ERM in patients. This mechanism was not directly evaluated in our cohort.

The present study has several limitations. Its retrospective design introduces selection bias and residual confounding. Treatment was not randomized and reflected real-world physician decision-making. Dexamethasone implant-treated eyes may therefore have differed from anti-VEGF-treated eyes in chronicity, inflammatory phenotype, pseudophakia, prior exposure, and follow-up feasibility. Although key measured covariates were adjusted, unmeasured confounding may remain. The 24-month administration count was ascertained after baseline and over the same window as ERM detection, so its association is noncausal and susceptible to reverse causation. ERM diagnosis on OCT may also be subjective, particularly when distinguishing early ERM from a thickened posterior hyaloid or subtle vitreoschisis.

The relatively small number of ERM events restricted the covariates that could be included in the primary model and required a parsimonious prespecified approach. Prior macular laser, lens status, prior vitrectomy, prior injection count, treatment-naive status, and individual anti-VEGF agent were therefore evaluated in secondary or sensitivity analyses. DRSS change was exploratory because it was post-treatment and may not have been uniformly available. Faricimab-specific conclusions are also limited by possible differences in real-world selection and follow-up compared with older agents.

Strengths include pretreatment OCT confirmation of ERM absence, evaluation of multiple contemporary intravitreal agents in one framework, assessment of ERM timing, and analysis of visual and anatomical associations. The models considered disease severity, prior retinal laser, 24-month administration count, and baseline VMI status. Based on these findings, we interpret treatment-emergent ERM as unlikely to represent a simple drug-specific adverse event. Instead, the observed pattern appears to reflect an interaction among diabetic retinal disease severity, VMI susceptibility, prior treatment/laser exposure, and intravitreal agent. This interpretation should be tested prospectively.

Taken together, the present study found that treatment-emergent ERM occurred in a clinically relevant proportion of DME eyes and was associated with poorer anatomical and visual outcomes. The higher adjusted ERM signal with dexamethasone implant should be interpreted in the context of nonrandomized treatment selection and baseline disease differences. Baseline VMI abnormality, severe NPDR/PDR, prior PRP, and higher baseline CST were important predictors within our cohort. No significant differences were detected among conventional anti-VEGF agents, and the lower numerical signal with faricimab remained exploratory. These conclusions are limited to the present observational cohort and do not establish causal or molecule-specific biological effects.

## 4. Materials and Methods

### 4.1. Study Design and Setting

This was a retrospective, observational, nonrandomized, real-world, OCT-based comparative study evaluating the incidence, timing, predictors, and clinical impact of treatment-emergent ERM in eyes with DME treated with intravitreal pharmacotherapy. The study was conducted across four eye care networks. Eligible index-treatment dates ranged from January 2023 through April 2024, and 24-month follow-up therefore extended through April 2026. The study adhered to the tenets of the Declaration of Helsinki and was approved by the Institutional Ethics Committee.

### 4.2. Study Population

Medical records and OCT images of patients with center-involving DME who received intravitreal treatment were screened. The index date was the first administration, within the study window, of the study agent that was maintained throughout the 24-month observation period. Treatment was not randomized or protocol assigned. Eyes were classified according to the intravitreal agent received in routine clinical care: dexamethasone implant, aflibercept, ranibizumab, bevacizumab, or faricimab.

Both treatment-naive and previously treated eyes were eligible. Eyes with previous intravitreal treatment exposure were not excluded, but the number of prior injections before the index date was recorded and analyzed as a potential confounder. One or both eyes of eligible patients were included. Because some patients contributed both eyes, the primary analysis was performed at the eye level, and sensitivity analysis was performed to account for inter-eye correlation using clustered standard errors at the patient level and/or by excluding fellow eyes where appropriate.

### 4.3. Inclusion and Exclusion Criteria

Eyes were included if they had center-involving DME, age 18 years or older at treatment initiation, treatment with one of the study intravitreal agents, baseline OCT confirming absence of clinically evident macular ERM, baseline best-corrected visual acuity (BCVA) and central subfield thickness (CST) data, and follow-up OCT data available through 24 months.

Eyes were excluded if they had pre-existing macular ERM at baseline, previous ERM peeling or macular surgery, coexisting retinal pathology likely to affect macular anatomy or visual outcome, poor-quality OCT images preventing reliable VMI assessment, incomplete required clinical/OCT data, inadequate 24-month follow-up, retinal laser or vitrectomy during follow-up, or switching from the index intravitreal agent during follow-up. Prior PRP, prior macular laser, prior intravitreal injections, lens status, and prior vitrectomy before the index date were not automatic exclusion criteria and were recorded as potential confounders.

### 4.4. Treatment Groups and Data Collection

Eyes were classified into five groups according to the index intravitreal agent maintained over 24 months. Agent selection was physician directed and reflected routine clinical judgment, including DME phenotype and chronicity, previous response, ocular status, anticipated follow-up feasibility, and affordability where relevant. No included eye switched intravitreal agents. For each eye, the total number of intravitreal procedures during the fixed 24-month period was recorded as the 24-month intravitreal administration count: implants for dexamethasone and injections for anti-VEGF/faricimab. This variable represents procedure frequency over time, not drug dose, and one implant was not considered pharmacologically equivalent to one anti-VEGF injection. Prior intravitreal injection exposure before the index date was recorded where available.

Baseline variables included age, sex, laterality, diabetes duration, DME duration, treatment-naive or previously treated status, lens status, prior cataract surgery, prior vitrectomy, prior PRP, prior macular laser, prior intravitreal injections, BCVA, CST, DR severity, PDR status, VMI status, and intraretinal or subretinal fluid. BCVA was recorded at baseline before the index administration and at 6, 12, 18, and 24 months. Snellen acuity was converted to logMAR for analysis, and ETDRS letter equivalents were calculated for presentation.

### 4.5. BCVA, OCT Assessment, and Definition of ERM

The baseline SD-OCT scan was the last eligible scan obtained before the index administration and was reviewed to confirm the absence of macular ERM and to grade other VMI features. OCT was then assessed at 6, 12, 18, and 24 months. Platforms included Zeiss Cirrus (Carl Zeiss Meditec, Jena, Germany), Heidelberg Spectralis (Heidelberg Engineering GmbH, Heidelberg, Germany) and/or Topcon systems (Topcon Corporation, Tokyo, Japan) according to center availability. At each time point, CST and macular morphology, including intraretinal/subretinal fluid and VMI configuration, were recorded. CST was taken from the automated output with manual correction when segmentation error was evident.

ERM was defined as a hyperreflective preretinal membrane on the inner retinal surface on OCT, with or without retinal surface wrinkling, foveal contour alteration, or tractional thickening. Treatment-emergent ERM was defined as the new appearance of macular ERM during follow-up in an eye documented to have no macular ERM at baseline. The time to ERM development was defined as the interval between the index treatment date and the first follow-up OCT scan showing ERM.

Baseline vitreomacular interface status was categorized as no abnormality, vitreomacular adhesion, vitreomacular traction, posterior vitreous detachment where identifiable, or other non-ERM vitreomacular interface abnormality. OCT grading was performed by two retina specialists masked to treatment group where feasible, with disagreements resolved by consensus and/or adjudication.

### 4.6. DR Severity and Outcomes

DR severity was recorded from clinical examination, fundus photography, fluorescein angiography where available, and medical record documentation. Eyes were categorized as mild non-proliferative diabetic retinopathy (NPDR), moderate NPDR, severe NPDR, or proliferative diabetic retinopathy (PDR). Where Diabetic Retinopathy Severity Scale (DRSS) grading was available, baseline and follow-up scores were recorded. A two-step or greater improvement in DRSS was considered clinically meaningful regression, and a two-step or greater worsening was considered progression. Because DRSS change is a post-treatment variable and may lie on the causal pathway between treatment type and ERM development, it was not included in the primary multivariable model and was analyzed separately as an exploratory outcome.

The primary outcome was treatment-emergent ERM formation over 24 months. Secondary outcomes included time to first ERM detection, ERM incidence by treatment group, comparison between dexamethasone implant and pooled non-dexamethasone therapy, comparison among individual anti-VEGF agents, associations with baseline severity and prior treatments, changes in BCVA and CST, final BCVA and CST according to ERM status, and requirement for pars plana vitrectomy or ERM peeling.

### 4.7. Statistical Analysis

Data were analyzed using SPSS version 30.0 (IBM Corp., Armonk, NY, USA). Continuous variables were expressed as mean ± standard deviation or median with interquartile range, depending on distribution. Categorical variables were expressed as frequencies and percentages. Normality was assessed using the Shapiro–Wilk test. Baseline continuous variables were compared across treatment groups using one-way analysis of variance or the Kruskal–Wallis test, as appropriate. Categorical variables were compared using the chi-square test or Fisher’s exact test.

Crude ERM incidence was calculated for each treatment group. Overall comparison across all treatment groups was performed using chi-squared analysis. The prespecified primary crude comparison was dexamethasone implant versus pooled non-dexamethasone therapy and was analyzed using Fisher’s exact test. Relative risk and odds ratio with 95% confidence intervals were calculated. Pairwise comparisons between dexamethasone implant and individual agents were also considered and were adjusted for multiple comparisons.

Factors associated with treatment-emergent ERM were first evaluated using univariate logistic regression. Clinically relevant variables included age, sex, baseline BCVA, baseline CST, baseline DR severity, PDR status, prior PRP, prior macular laser, lens status, prior vitrectomy, treatment-naive versus previously treated status, prior injection count, the 24-month intravitreal administration count, baseline VMI abnormality, and treatment category.

To reduce overfitting, the primary multivariable logistic model was restricted to prespecified covariates: treatment category, baseline VMI abnormality, severe NPDR/PDR, prior PRP, baseline CST per 100 µm, and 24-month intravitreal administration count. The administration count was entered as a continuous fixed-period exposure adjustment (per administration). Because it was accumulated after treatment initiation, it was not interpreted as a baseline predictor, drug-dose measure, or causal effect. Secondary or sensitivity models evaluated prior macular laser, lens status, prior vitrectomy, treatment-naive status, prior injection count, individual anti-VEGF agent, and DRSS change.

Time to ERM formation was evaluated using Kaplan–Meier analysis, with eyes not developing ERM censored at the last follow-up visit; ERM-free survival was compared using the log-rank test. The post-baseline 24-month administration count was not entered into time-to-event modeling. Longitudinal BCVA and CST outcomes were analyzed using linear mixed-effects models with fixed effects for time, treatment group, ERM status, and relevant interaction terms. All *p* values were two-sided, and *p* < 0.05 was considered statistically significant.

## 5. Conclusions

In the present study, treatment-emergent ERM in DME was associated with baseline disease severity, VMI status, prior retinal laser exposure, and intravitreal treatment category. Dexamethasone implant-treated eyes had the highest adjusted ERM signal, whereas no significant differences were detected among conventional anti-VEGF agents. Faricimab had the lowest numerical ERM rate, but this observation was exploratory. ERM formation was associated with poorer anatomical and visual outcomes. These findings support OCT-based VMI monitoring during DME treatment while emphasizing that prospective studies are required to distinguish treatment effects from disease severity and treatment-selection factors.

## Figures and Tables

**Table 1:** Baseline demographic and ocular characteristics by treatment group.

Characteristic	Dexamethasone Implant (*n* = 75)	Aflibercept (*n* = 67)	Ranibizumab (*n* = 104)	Bevacizumab (*n* = 108)	Faricimab (*n* = 69)	*p* Value
Age, years	62.3 ± 8.7	61.8 ± 9.1	60.4 ± 9.6	59.1 ± 9.4	60.9 ± 9.0	0.184
Male sex, *n* (%)	42 (56.0)	38 (56.7)	59 (56.7)	61 (56.5)	38 (55.1)	0.999
Baseline BCVA, logMAR	0.72 ± 0.24	0.66 ± 0.22	0.62 ± 0.21	0.65 ± 0.23	0.62 ± 0.22	0.018
Baseline BCVA, ETDRS letters	49.0 ± 12.0	52.0 ± 11.0	54.0 ± 10.5	52.5 ± 11.5	54.0 ± 11.0	0.018
Baseline CST, µm	520.1 ± 81.8	479.3 ± 85.5	451.5 ± 84.8	470.2 ± 85.8	461.7 ± 81.5	<0.001
DME duration, months	22.8 ± 13.8	19.7 ± 12.5	16.8 ± 11.9	15.9 ± 11.4	17.5 ± 12.1	0.003
Severe NPDR or PDR, *n* (%)	60 (80.0)	47 (70.1)	68 (65.4)	76 (70.4)	48 (69.6)	0.214
PDR, *n* (%)	27 (36.0)	20 (29.9)	25 (24.0)	27 (25.0)	14 (20.3)	0.173
Prior PRP, *n* (%)	39 (52.0)	28 (41.8)	35 (33.7)	41 (38.0)	21 (30.4)	0.040
Prior macular laser, *n* (%)	31 (41.3)	22 (32.8)	29 (27.9)	31 (28.7)	14 (20.3)	0.057
Pseudophakic, *n* (%)	44 (58.7)	28 (41.8)	38 (36.5)	37 (34.3)	24 (34.8)	0.002
Prior vitrectomy, *n* (%)	4 (5.3)	3 (4.5)	4 (3.8)	4 (3.7)	3 (4.3)	0.984
Prior intravitreal injections, *n*	2.5 ± 1.9	2.3 ± 1.8	1.6 ± 1.4	1.5 ± 1.3	2.0 ± 1.7	<0.001
Treatment-naive eyes, *n* (%)	12 (16.0)	11 (16.4)	27 (26.0)	30 (27.8)	17 (24.6)	0.220
Previously treated eyes, *n* (%)	63 (84.0)	56 (83.6)	77 (74.0)	78 (72.2)	52 (75.4)	
Baseline VMI abnormality *, *n* (%)	29 (38.7)	19 (28.4)	25 (24.0)	27 (25.0)	14 (20.3)	0.062

* Baseline VMI abnormality included VMA, VMT, or other non-ERM vitreomacular interface abnormality.

**Table 2:** Treatment exposure and DRSS change by treatment group.

Variable	Dexamethasone Implant (*n* = 75)	Aflibercept (*n* = 67)	Ranibizumab (*n* = 104)	Bevacizumab (*n* = 108)	Faricimab (*n* = 69)	*p* Value
24-month intravitreal administration count	4.8 ± 1.9 implants	7.2 ± 1.4 injections	8.8 ± 1.4 injections	9.5 ± 1.4 injections	6.8 ± 1.4 injections	<0.001
Prior injections before index	2.5 ± 1.9	2.3 ± 1.8	1.6 ± 1.4	1.5 ± 1.3	2.0 ± 1.7	<0.001
≥2-step DRSS improvement, *n* (%)	5 (6.7)	15 (22.4)	25 (24.0)	18 (16.7)	19 (27.5)	0.008 †
Stable DRSS, *n* (%)	56 (74.7)	47 (70.1)	72 (69.2)	79 (73.1)	47 (68.1)	
≥2-step DRSS worsening, *n* (%)	14 (18.7)	5 (7.5)	7 (6.7)	11 (10.2)	3 (4.3)	

† *p* value refers to the overall distribution of DRSS improvement, stability, and worsening. DRSS change was exploratory and was not included in the primary multivariable model. The 24-month intravitreal administration count is a procedure-frequency measure (implants or injections), not a drug-dose equivalence.

**Table 3:** Incidence of treatment-emergent ERM by treatment group.

Treatment Group	Eyes, *n*	ERM Events, *n*	ERM Incidence (95% CI)
Dexamethasone implant	75	12	16.0% (9.4–25.9)
Aflibercept	67	5	7.5% (3.2–16.3)
Ranibizumab	104	8	7.7% (3.9–14.4)
Bevacizumab	108	8	7.4% (3.8–13.9)
Faricimab	69	3	4.3% (1.5–12.0)
Overall	423	36	8.5% (6.2–11.6)
Overall five-group comparison			χ^2^ = 7.29; df = 4; *p* = 0.121
Dexamethasone implant vs. pooled non-dexamethasone	75 vs. 348	12 vs. 24	RR = 2.32; OR = 2.57 (1.22–5.41); Fisher *p* = 0.020

CI = confidence interval; ERM = epiretinal membrane; RR = relative risk; OR = odds ratio. Incidence CIs are Wilson intervals.

**Table 4:** Pairwise and anti-VEGF subgroup comparisons.

Comparison	ERM Incidence	Odds Ratio (95% CI)	Unadjusted *p*	Bonferroni-Adjusted *p*
**Dexamethasone implant vs. Aflibercept**	16.0% vs. 7.5%	2.36 (0.79–7.10)	0.130	0.521
**Dexamethasone implant vs. Ranibizumab**	16.0% vs. 7.7%	2.29 (0.88–5.91)	0.096	0.382
**Dexamethasone implant vs. Bevacizumab**	16.0% vs. 7.4%	2.38 (0.92–6.15)	0.091	0.364
**Dexamethasone implant vs. Faricimab**	16.0% vs. 4.3%	4.19 (1.13–15.55)	0.028	0.114
**Aflibercept vs. ranibizumab vs. bevacizumab**	7.5% vs. 7.7% vs. 7.4%	Not applicable	0.997	Not applicable
**Aflibercept vs. ranibizumab vs. bevacizumab vs. faricimab**	7.5% vs. 7.7% vs. 7.4% vs. 4.3%	Not applicable	0.831	Not applicable

Pairwise comparisons were exploratory. Bonferroni-adjusted *p* values are shown for dexamethasone implant versus individual agents.

**Table 5:** Univariate analysis of clinically relevant factors associated with treatment-emergent ERM.

Variable	Comparison/Unit	Univariate OR (95% CI)	*p* Value	Primary Model Status
**Dexamethasone implant**	vs. pooled non-dexamethasone	2.57 (1.22–5.41)	0.020	Included
**Age**	per year	1.02 (0.99–1.06)	0.180	Not included
**Male sex**	male vs. female	1.05 (0.53–2.09)	0.880	Not included
**Baseline BCVA**	per 0.1 logMAR worse	1.21 (1.01–1.45)	0.041	Assessed; not included in primary model
**Baseline CST**	per 100 µm increase	1.73 (1.17–2.55)	0.006	Included
**DME duration**	per 6-month increase	1.11 (0.99–1.25)	0.080	Sensitivity
**Severe NPDR/PDR**	vs. mild/moderate NPDR	3.59 (1.23–10.48)	0.019	Included
**PDR**	vs. NPDR	2.10 (1.03–4.29)	0.041	Assessed; not included with severe NPDR/PDR
**Prior PRP**	yes vs. no	5.48 (2.52–11.91)	<0.001	Included
**Prior macular laser**	yes vs. no	3.27 (1.63–6.55)	0.001	Sensitivity
**Pseudophakia**	yes vs. no	1.35 (0.68–2.68)	0.390	Sensitivity
**Prior vitrectomy**	yes vs. no	Sparse events	0.380	Descriptive/sensitivity only
**Prior injection count**	per prior injection	1.10 (0.95–1.27)	0.205	Sensitivity
**24-month intravitreal administration count**	per administration	1.16 (1.01–1.33)	0.039	Exploratory post-baseline exposure adjustment
**Baseline VMI abnormality**	yes vs. no	7.89 (3.73–16.68)	<0.001	Included
**≥2-step DRSS improvement**	yes vs. no	0.82 (0.33–2.03)	0.690	Exploratory; post-treatment variable

All clinically relevant confounders were assessed at the univariate stage. DRSS improvement was analyzed separately because it is a post-treatment variable.

**Table 6:** Primary multivariable logistic regression model for treatment-emergent ERM.

Covariate	Adjusted OR (95% CI)	*p* Value	Comment
Dexamethasone implant vs. pooled non-dexamethasone	2.24 (1.02–4.92)	0.044	Primary exposure
Baseline VMI abnormality	6.10 (2.80–13.31)	<0.001	Strongest baseline predictor
Severe NPDR/PDR	2.75 (1.02–7.40)	0.046	Baseline disease severity
Prior PRP	3.95 (1.78–8.77)	0.001	Marker of ischemic/severe DR
Baseline CST per 100 µm	1.55 (1.05–2.29)	0.028	Anatomical burden
24-month intravitreal administration count	1.07 (0.90–1.28)	0.440	Post-baseline exposure adjustment; noncausal

The primary model was restricted to prespecified covariates to reduce overfitting due to the limited number of ERM events. Prior macular laser, lens status, prior vitrectomy, and prior injection count were evaluated in secondary or sensitivity models.

**Table 7:** Visual and anatomical outcomes according to ERM status.

Outcome	No Treatment-Emergent ERM (*n* = 387)	Treatment-Emergent ERM (*n* = 36)	Unadjusted *p*	Adjusted Estimate
Baseline BCVA, logMAR	0.64 ± 0.22	0.75 ± 0.25	0.023	Adjusted in outcome model
Final BCVA, logMAR	0.50 ± 0.21	0.69 ± 0.26	<0.001	+0.09 logMAR worse final BCVA (95% CI 0.04–0.14); *p* < 0.001
BCVA gain, ETDRS letters	+7.1 ± 6.0	+2.8 ± 5.1	<0.001	Adjusted mean difference 4.3 letters; *p* < 0.001
Baseline CST, µm	470.9 ± 84.7	513.2 ± 92.4	0.006	Adjusted in outcome model
Final CST, µm	340.1 ± 72.0	424.6 ± 89.0	<0.001	+48.6 µm higher final CST (95% CI 23.9–73.2); *p* < 0.001
CST reduction, µm	130.8 ± 76.0	88.6 ± 70.0	0.004	Adjusted mean difference 42.2 µm; *p* = 0.004

Adjusted estimates control for baseline BCVA/CST, treatment group, baseline DR severity, prior PRP, prior macular laser, and baseline VMI abnormality.

**Table 8:** Sensitivity and secondary analyses details.

Sensitivity/Secondary Model	Purpose	Result for Dexamethasone Implant Effect	Interpretation
Primary parsimonious model	Main adjusted analysis	Adjusted OR 2.24 (1.02–4.92); *p* = 0.044	DEX remained independently associated with ERM
Model adding prior macular laser	Assess laser confounding	Adjusted OR 2.08 (0.98–4.43); *p* = 0.060	Direction preserved; borderline
Model adding lens status	Assess pseudophakia/steroid selection bias	Adjusted OR 2.26 (1.03–4.97); *p* = 0.043	Effect retained
Model adding prior injection count	Assess prior treatment history	Adjusted OR 2.18 (1.00–4.76); *p* = 0.050	Borderline but consistent
Excluding prior vitrectomy eyes	Avoid altered VMI biology	Adjusted OR 2.36 (1.05–5.31); *p* = 0.038	Effect retained
Excluding baseline VMA/VMT eyes	Evaluate de novo ERM in structurally normal VMI	Adjusted OR 1.72 (0.73–4.07); *p* = 0.210	Effect attenuated; VMI status is important
Treatment-naïve subgroup	Reduce prior-treatment bias	Adjusted OR 2.10 (0.89–4.95); *p* = 0.090	Direction preserved; underpowered
Five-category treatment model	Individual-agent comparison	DEX highest adjusted odds; anti-VEGF agents not significantly different	Supports DEX vs. non-DEX primary framing
DRSS improvement exploratory model	Assess disease-modification hypothesis	≥2-step DRSS improvement OR 0.83 (0.39–1.78); *p* = 0.620	DRSS change exploratory; not part of primary model

Sensitivity analyses were performed to test whether the primary finding was robust to key clinical assumptions and potential confounders.

## Data Availability

The original contributions presented in the study are included in the article, further inquiries can be directed to the corresponding author. De-identified individual-level data underlying the results of this study, together with a data dictionary, may be made available by the corresponding author upon reasonable request, subject to approval by the participating institutions and ethics committee and execution of an appropriate data-use agreement.

## Abbreviations

BCVA, best-corrected visual acuity; CI, confidence interval; CST, central subfield thickness; DME, diabetic macular edema; DR, diabetic retinopathy; DRSS, Diabetic Retinopathy Severity Scale; ERM, epiretinal membrane; NPDR, non-proliferative diabetic retinopathy; OCT, optical coherence tomography; OR, odds ratio; PDR, proliferative diabetic retinopathy; PRP, panretinal photocoagulation; VEGF, vascular endothelial growth factor; VMA, vitreomacular adhesion; VMI, vitreomacular interface; VMT, vitreomacular traction.
